# Dimethyl Fumarate Attenuates Lymphocyte Infiltration and Reduces Infarct Size in Experimental Stroke

**DOI:** 10.3390/ijms242115540

**Published:** 2023-10-24

**Authors:** Michael K. Schuhmann, Friederike Langhauser, Lena Zimmermann, Maximilian Bellut, Christoph Kleinschnitz, Felix Fluri

**Affiliations:** 1 Department of Neurology, University Hospital Würzburg, Josef-Schneider Strasse 11, 97080 Würzburg, Germany; schuhmann_m@ukw.de (M.K.S.); papp_l@ukw.de (L.Z.); bellut_m@ukw.de (M.B.); 2 Department of Neurology and Center for Translational Neuro- and Behavioral Sciences (C-TNBS), University Medicine Essen, 45147 Essen, Germany; friederike.langhauser@uk-essen.de (F.L.); christoph.kleinschnitz@uk-essen.de (C.K.)

**Keywords:** experimental stroke, transient middle cerebral artery occlusion model, dimethyl fumarate, cerebral inflammation

## Abstract

Ischemic stroke is associated with exacerbated tissue damage caused by the activation of immune cells and the initiation of other inflammatory processes. Dimethyl fumarate (DMF) is known to modulate the immune response, activate antioxidative pathways, and improve the blood–brain barrier (BBB) after stroke. However, the specific impact of DMF on immune cells after cerebral ischemia remains unclear. In our study, male mice underwent transient middle cerebral artery occlusion (tMCAO) for 30 min and received oral DMF (15 mg/kg) or a vehicle immediately after tMCAO, followed by twice-daily administrations for 7 days. Infarct volume was assessed on T2-weighted magnetic resonance images on days 1 and 7 after tMCAO. Brain-infiltrating immune cells (lymphocytes, monocytes) and microglia were quantified using fluorescence-activated cell sorting. DMF treatment significantly reduced infarct volumes and brain edema. On day 1 after tMCAO, DMF-treated mice showed reduced lymphocyte infiltration compared to controls, which was not observed on day 7. Monocyte and microglial cell counts did not differ between groups on either day. In the acute phase of stroke, DMF administration attenuated lymphocyte infiltration, probably due to its stabilizing effect on the BBB. This highlights the potential of DMF as a therapeutic candidate for mitigating immune cell-driven damage in stroke.

## 1. Introduction

Ischemic stroke results from thrombotic or embolic occlusion of a cerebral artery, leading to a disruption in blood supply and glucose delivery and ultimately causing brain cell injury [1]. Subsequent to ischemic neuronal damage, various factors trigger an inflammatory cascade [2,3]. Microglial cells, the resident macrophages of the brain, are activated within minutes after the onset of cerebral ischemia and release proinflammatory agents such as tumor necrosis factor alpha (TNF-α) or interleukin-1 beta (IL-1β) [4,5]. These proinflammatory agents contribute to the breakdown of the blood–brain barrier (BBB), allowing blood-borne immune cells such as neutrophils, monocytes, and T-cells to invade the ischemic brain [6,7]. As neuronal tissue damage progresses, damage-associate molecular patterns (DAMPs) are produced, which amplify neuroinflammation through activating additional microglial cells and attracting leukocytes from the peripheral blood [2,3]. Therefore, targeting molecules that alleviate leukocyte trafficking into the ischemic brain vasculature presents an attractive strategy for treating neuroinflammation.

Interestingly, dimethyl fumarate (DMF, the methyl ester of fumaric acid) and its active metabolite (monomethyl fumarate, MMF) act as an antioxidant and immunomodulator in animal models [8,9] and in patients with neurological diseases, such as multiple sclerosis [10,11]. DMF exerts an antiproliferative effect, probably linked to a transient elevation of free intracellular calcium [12], and shifts the T-cell response towards a Th2 cytokine profile [13,14], resulting in a reduction in CD4^+^ and CD8^+^ T-cells [13]. In animal stroke models, DMF reduced oxidative stress, proinflammatory cytokines, cerebral edema, and apoptosis through the nuclear factor erythroid 2-related factor 2 (Nrf2) pathway [15]. However, the effect of DMF on the infiltration of immune cells into the ischemic hemisphere has not been addressed. Accordingly, we investigated the impact of DMF on various types of immune cells in a murine stroke model. Through exploring the effects of DMF on infarct size, BBB function, and its potential to mitigate immune cell infiltration, we aimed to shed light on promising therapeutic avenues for targeting neuroinflammation in the context of ischemic stroke.

## 2. Results

### 2.1. Infarct Volumes Decreased after DMF Treatment

**Figure 1:**
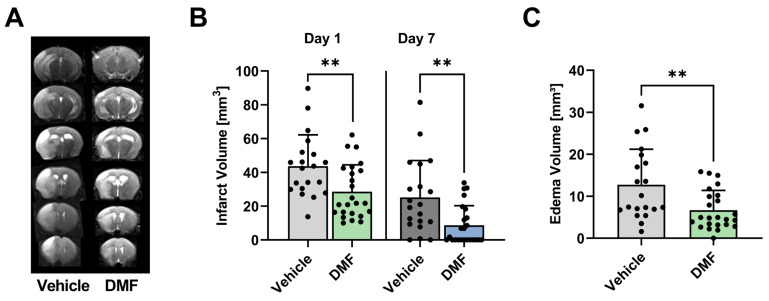
Infarct volume and cerebral edema of DMF- and vehicle-treated animals. (**A**) Representative T2w scans of a vehicle- and DMF-treated animal. (**B**) Infarct volumes of DMF-treated animals were significantly smaller in the acute phase of stroke, i.e., on days 1 and 7 compared to controls (n = 20–24/group). (**C**) Development of brain edema on day 1 after tMCAO decreased significantly after administration of DMF compared to vehicle (n = 20–24/group). ** *p* < 0.01; unpaired, two-tailed Student’s *t*-test.

In the first series of experiments, we assessed the infarct size and perifocal edema extent in DMF- and vehicle-treated animals (controls) following transient middle cerebral artery occlusion (tMCAO). Infarct volumes were measured using planimetry of T2-weighted (T2w) MRI scans (Figure 1A). After tMCAO, DMF-treated mice exhibited significantly smaller infarct volumes compared to control animals on day 1 (mean infarct volume 43.7 ± 18.5 mm^3^ vs. 28.6 ± 15.9 mm^3^; *p* < 0.01) and on day 7 (mean infarct volume 25.2 ± 21.9 mm^3^ vs. 8.7 ± 11.6 mm^3^; *p* < 0.01) (Figure 1B). Furthermore, DMF treatment significantly reduced the development of infarct-induced brain edema on day 1 after tMCAO compared to controls (Figure 1C).

### 2.2. DMF Treatment Was Associated with a Reduced Number of Lymphocytes within the Ischemic Hemisphere

**Figure 2:**
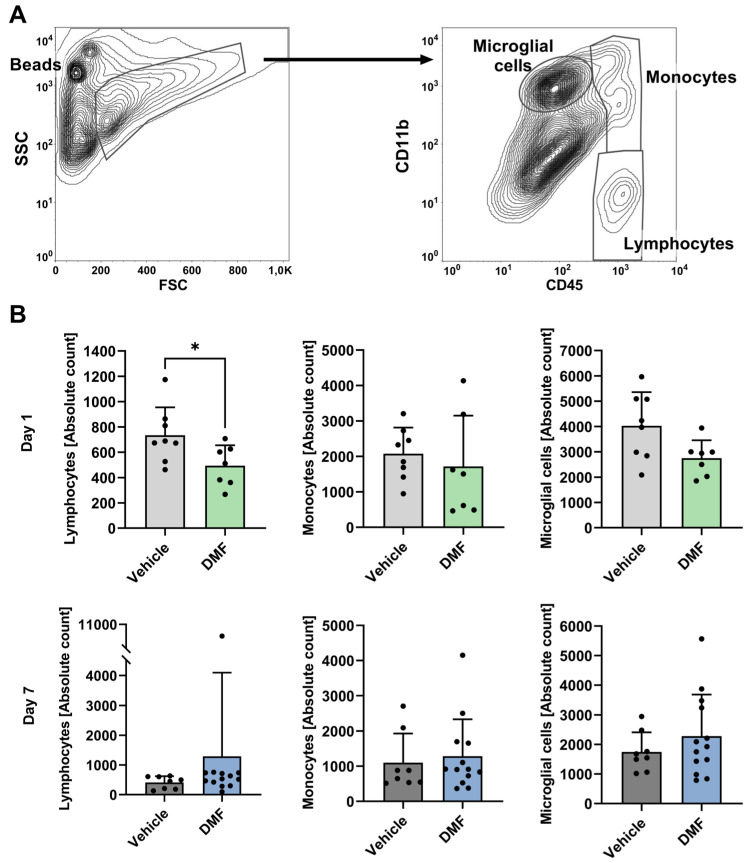
Immune cell recruitment to the ischemic brain hemisphere on days 1 and 7 after induction of tMCAO. Flow cytometric analysis of lymphocytes, monocytes, and microglial cells in the ischemic brains of vehicle- and DMF-treated animals. (**A**) Gating strategy for the flow cytometric evaluation of recruited immune cells. Representative contour plot refining the counting bead population and indicating a preselection of infiltrating immune cells into the ischemic hemisphere within the FSC and SSC plot (left). The gates defining the lymphocyte, monocyte, and microglial cell populations are shown within the CD45 and CD11b contour plot (right). (**B**) Absolute immune cell numbers within the ischemic brain on day 1 (n = 7–8/group) and day 7 after tMCAO (n = 8–13/group). Lymphocyte cell numbers were significantly reduced in DMF-treated animals compared to controls (vehicle) on day 1, but not on day 7 after tMCAO. Monocyte and microglial cell numbers did not differ between treatment groups on either day (day 1: n = 7–8/group; day 7: n = 8–13/group). * *p* < 0.05; two-tailed Mann–Whitney test.

We further investigated the impact of DMF administration on the number of microglial cells and invading lymphocytes and monocytes using fluorescence-activated cell sorting (FACS) analyses (Figure 2A). On day 1 after tMCAO, there was a non-significant reduction in microglial cells within the ischemic brain tissue of DMF-treated animals compared to the vehicle-treated group; in contrast, the number of invading lymphocytes was significantly lower in the DMF-treated mice (Figure 2B). There was no significant difference in the number of monocytes between DMF- and vehicle-treated animals on day 1 after tMCAO. On day 7 after tMCAO, there was no significant difference in the number of resident or invading immune cells between vehicle- and DMF-treated animals (Figure 2B).

## 3. Discussion

Inflammation has been recognized as a main contributor to the pathophysiology of ischemic stroke in humans as well as in animal models [16]. There is growing evidence that circulating immune cells are already involved at the onset of arterial occlusion, which finally results in the invasion of blood-borne immune cells into the ischemic brain and in an activation of brain-resident cells [17]. In this context, neutrophils, monocytes, and T-cells are the most important immune cells which invade into the infarcted brain area and thus play a crucial role in propagating cerebral tissue damage [16]. Of note, these immune cells are also engaged in the chronic phase of ischemic stroke. Hence, the modulation of inflammatory processes, in particular immune cells immediately after onset of stroke, may exert a protective effect on ischemic brain tissue and, therefore, would be a promising stroke therapy. One agent qualifying for such a modulator is DMF, which has antiproliferative properties [12] and affects T-cell response [13,14]. In the present study, we examined the effect of DMF on immune cells within the infarcted brain hemisphere of mice after a tMCAO of 30 min. We have demonstrated that oral administration of DMF to mice immediately after the induction of ischemic stroke, and thereafter twice daily for one week, resulted in (1) significantly less brain infarction (days 1 and 7) and cerebral edema (day 1) and (2) a decrease in lymphocyte counts in the ischemic brain on day 1 after intervention compared to controls.

DMF-treated mice exhibited significantly smaller infarct sizes, which differed by ~50% compared to controls on day 1 and even on day 7 after the induction of ischemic stroke. These findings are in line with a previous study that demonstrated a dose-dependent reduction in infarct size with DMF treatment [18]. Interestingly, the route of administration appears to play a crucial role in the effect of the drug. In mice subjected to tMCAO, oral pretreatment with DMF resulted in a decrease in infarct size compared to control mice [19], whereas there was no significant difference after intraperitoneal application [20], suggesting that the route of administration might be important for the drug effect. A significant decrease in infarct size after oral gavage of DMF has also been shown in other species, such as rats [21,22].

A further important aspect of our study is the impact of DMF on the integrity of the BBB. DMF-treated mice had significantly less perifocal edema on day 1 after induction of ischemic stroke compared to control mice. This indicates that DMF may protect the BBB. Our study corroborates data from other studies examining the effect of DMF on the formation of perifocal edema in a murine stroke model [18,20]. Kunze and coworkers reported that DMF had a protective impact on the tight junctions of the BBB [20]. Tight junctions, such as claudin-5, zonula occludens-1 (ZO-1), and occludin, form a serial zipper-like seal between adjacent endothelial cells; this allows brain capillaries to gatekeep the paracellular flux of molecules from the blood into the cerebral parenchyma [23]. When animals were pretreated with DMF, the amount of occludin and claudin-5 protein was significantly increased in the infarct core compared to controls; additionally, the subcellular localization of occludin and ZO-1 in capillaries of the perilesional area surrounding the infarction was nearly sustained in DMF-pretreated animals [20]. Moreover, DMF has been shown to interfere with the activation of matrix metalloproteinase (MMP), which plays a role in the opening of the BBB during cerebral ischemia [23]. In this context, DMF reduced the number of cells that showed MMP activity within the ischemic brain tissue (most often neurons but also endothelial cells) [20]. These observations further underline the BBB-stabilizing property of DMF that contributes to decreased edema formation after stroke.

A key finding of our study is a notable decrease in lymphocyte infiltration into the ischemic hemisphere on day 1 after tMCAO in DMF-treated mice, as assessed via flow cytometry. The number of monocytes and microglial cells was lower among DMF-treated mice compared to controls. However, the difference did not achieve statistical significance, which is probably due to the wide confidence interval and the relatively small number of animals, which is a limitation of our study. Importantly, recently published studies found a significant reduction in microglia activation in infarcted brains after the DMF treatment of mice [19,24]. Furthermore, we have no independent verification of the presented flow cytometric data with an immunohistochemistry approach. Additionally, if cells are disproportionately damaged or lysed in the experimental groups for flow cytometry, quantitative comparisons may be compromised.

In addition, one might argue that a reduced lymphocyte count in the ischemic brain reflects DMF-induced lymphopenia in the peripheral blood, as can occur in patients with multiple sclerosis [10,11,25]. However, lymphopenia after DMF treatment has not been reported in the short term but rather within 6 to 12 months after therapy initiation [25]. Interestingly, some studies revealed that DMF may also alter the number and activity of immune cells within the cerebral parenchyma through modulating transendothelial migration of immune cells through the BBB [26,27]. Studies have shown that DMF downregulates intercellular adhesion molecule 1 and vascular cell adhesion molecule, as well as E-selectin, leading to a decrease in the migration of T-cells across the BBB [28,29,30,31]. The underlying mechanisms could involve the activation of Nrf2 [27,32], which subsequently regulates many molecules with antioxidative effects and downregulates inflammatory cascades following stroke.

In summary, our results demonstrate that oral administration of DMF in the acute phase of ischemic stroke attenuates recruitment of lymphocytes in the ischemic brain, which might be due to a stabilizing effect of DMF on BBB function.

## 4. Materials and Methods

### 4.1. Animals

We used a total of 74 male 6–8-week-old C57Bl/6 mice (Charles River Laboratories, Sulzfeld, Germany). Out of them, 58 animals, i.e., 28 DMF-treated animals and 30 vehicle-treated mice (controls), were initially allocated to the MRI group. MRI was performed on day 1 and day 7 after tMCAO.

Out of these 58 animals, 1 DMF-treated animal showed a substantial loss of blood during surgery; 9 vehicle-treated animals and 2 DMF-treated animals were euthanized due to severe impairment between induction of tMCAO and MRI examination on day 7 and thus were excluded from the study. One animal from each group did not exhibit an infarction on T2 weighted MRI scans on day 1 and on day 7. Finally, 24 DMF-treated and 20 vehicle-treated animals showed an MRI-confirmed infarction on day 1. Only animals in which two MRI examinations were completed have been used for the assessment of infarct volume. A predefined subgroup of these animals was used for FACS-analysis on day 7. We used another 16 animals (8 DMF- and 8 vehicle-treated animals) for FACS-analysis on day 1 after tMCAO. Out of them, 1 DMF-treated animal died and was excluded from the study.

All experiments were approved by the government of Lower Franconia (Bavaria/Germany) and were in accordance with the recommendations for research in experimental stroke studies [33] and the current Animal Research: Reporting of In Vivo Experiments guidelines (http://www.Nc3rs.org/ARRIVE accessed on 27 June 2023).

### 4.2. Administration of Dimethyl Fuarate (DMF)

DMF was dissolved in water containing 0.08% methocel and applied via oral gavage (15 mg/kg body weight) immediately after tMCAO, and thereafter twice daily for 7 days [8]. Oral gavage with an equal volume of water/0.08% methocel served as a control (vehicle).

### 4.3. Transient Middle Cerebral Artery Occlusion (tMCAO) Model

Focal cerebral ischemia was induced via 30-min transient middle cerebral artery occlusion (tMCAO) as previously described [34]: Briefly, animals were anesthetized with 2.5% isoflurane (Abbott, Wiesbaden, Germany). Next, a midline skin incision was cut in the neck and then the proximal common carotid artery and the external carotid artery were exposed and ligated. Thereafter, a silicon rubber-coated 6.0 nylon monofilament (6023910PK10 (Doccol Corporation, Redlands, CA, USA)) was inserted in the right internal carotid artery and advanced until a slight resistance was recognized, indicating the origin of the right middle cerebral artery (MCA), which results in an occlusion of the MCA. The suture was then left in this position for 30 min. The operators were blinded to the treatment groups. The maximal time of surgery per animal did not exceed 15 min.

Exclusion criteria were (1) subarachnoid hemorrhage or bleeding into the brain parenchyma (as visually determined during brain sampling); (2) surgery time > 15 min; (3) no visible infarction on T2 weighted MRI scans (day 1 and day 7); and (4) substantial loss of blood during surgery. Animals were checked regarding their health state during all experiments. Euthanasia of severely impaired post-stroke animals was performed using a deep isoflurane anesthesia.

### 4.4. Determination of Infarct Volume and Edema

To assess intra-individual temporal evolution of infarct volume, magnetic resonance imaging (MRI) was performed on a 3.0-T scanner (MAGNETOM Trio, Siemens, Munich, Germany) on days 1 and 7 after tMCAO, using T2w turbo spin-echo sequences (echo time, 105 ms; repetition time, 2100 ms). Lesion volumes were determined on T2w scans using ImageJ Analysis Software 1.45s (National Institutes of Health, Bethesda, MD, USA. http://rsb.info.nih.gov/ij/ (accessed on 29 October 2011)). The hyperintense (uncorrected) lesion and the remaining ipsilesional hemisphere as well as the contralesional hemisphere on each scan (1 mm thick) were traced manually and the areas were then summed and multiplied by the slice thickness and finally corrected for edema as proposed by Gerriets and coworkers [35]. Their proposed calculation of edema correction is based on the following three assumptions: (1) the compression of the contralateral hemisphere is similar to the compression of the whole healthy brain tissue, whereas the lesion is not compressed; (2) the contralateral hemisphere is compressed to the same degree as the ipsilesional, i.e., infarcted, area; and (3) extension of the volume occurs only within the lesion, not in the unaffected tissue. Taken together, this finally results in the following equation: LV^u^ = HVc + HVi − (HVc + Hvi − LV^u^) × (HVc + Hvi)/2 × HV_c_, where LV^c^ and LV^u^ indicate edema-corrected and uncorrected lesion volume, and HVc and Hvi indicate volumes of the contralateral and ipsilateral (lesioned) hemisphere [35]. Brain edema results computationally from the subtraction of LV^u^ from LV^c^ [35].

### 4.5. FACS Analyses

Brains from nonperfused mice were collected for the purification of brain-infiltrating cells. The right, i.e., lesioned, hemisphere was separated from the left hemisphere; only the right hemisphere was used for FACS analyses. The whole right hemisphere encompassing the infarction was cut into pieces and further mechanically homogenized in PBS, then layered on a 30–50% Percoll (Sigma-Aldrich, Taufkirchen, Germany) density gradient and continuously centrifuged for 30 min at 2.500 r.p.m. Mononuclear cells were isolated at the interphase. After isolation, cells were washed and resuspended in an appropriate buffer for further analysis. For the quantification of cells isolated from the ischemic hemisphere, Calibrite 3 Beads (BD Biosciences, Heidelberg, Germany) were added to freshly isolated brain-infiltrating immune cells before washing and staining with anti-CD45-FITC and anti-CD11b-PE antibodies (BD Biosciences, Heidelberg, Germany). Samples were analyzed using a FACS Calibur (BD Biosciences, Heidelberg, Germany) and evaluated through the Software FlowJo V10.8.1 (TreeStar, Ashland, OR, USA) [36].

### 4.6. Statistical Analyses

For statistical analysis, the GraphPad Prism v9.3.1 software package (GraphPad Software, La Jolla, CA, USA) was used. All results are presented as mean ± SD. Statistical analysis comparing two groups (vehicle vs. DMF) was performed using the two-tailed Student’s *t*-test or Mann–Whitney test. *p*-values < 0.05 were considered significant with * *p* < 0.05, ** *p* < 0.01, and *** *p* < 0.001.

## Data Availability

The analyzed data sets generated during the study are available from the corresponding author on reasonable request.
